# Two Insects, Two Bites, One Patient: A Lyme Disease and Jamestown Canyon Co-infection

**DOI:** 10.7759/cureus.40222

**Published:** 2023-06-10

**Authors:** Nicholas S Weiler, Eric Niendorf, Igor Dumic

**Affiliations:** 1 Research, Mayo Clinic Health Systems, Eau Claire, USA; 2 Radiology, Mayo Clinic, Eau Claire, USA; 3 Hospital Medicine, Mayo Clinic, Eau Claire, USA

**Keywords:** vector borne disease, meningitis, co-infection, lyme disease, jamestown canyon virus

## Abstract

Lyme disease (LD) is the most common tick-borne illness across the United States, caused by the bacterium *Borrelia burgdorferi sensu lato* and transmitted to humans by the bite of infected *Ixodes* ticks. Jamestown Canyon Virus (JCV) is an emerging mosquito-borne pathogen found mostly in the upper Midwest and Northeastern United States. Co-infection between these two pathogens has not been previously reported since it would require the host to be bitten by the two infected vectors at the same time. We report a 36-year-old man who presented with erythema migrans and meningitis. While erythema migrans is a pathognomonic sign of early localized Lyme disease, Lyme meningitis does not occur in this stage but in the early disseminated stage. Furthermore, CSF tests were not supportive of neuroborreliosis, and the patient was ultimately diagnosed with JCV meningitis. We review JCV infection, LD, and this first reported co-infection to illustrate the complex interaction between different vectors and pathogens and to emphasize the importance of considering co-infection in people who live in vector-endemic areas.

## Introduction

Lyme disease (LD) is the most common tick-borne illness across the United States [1]. Approximately 30,000 cases of LD are reported to the Centers for Disease Control and Prevention (CDC) each year, but it is estimated that 476,000 people may contract LD each year. With climate change, it is predicted that the incidence of LD will increase by at least 20% in the coming decades [2]. In the United States, LD is caused by the bacterium *Borrelia burgdorferi* and is delivered through the bite of *Ixodes* ticks. Untreated LD progresses through three stages after an incubation period of about three to 30 days. In the first stage, erythema migrans (EM) develops at the site of infection most commonly five to seven days following the tick bite [3]. This is the stage at which the disease is most easily identified and treated before it disseminates. The infection progresses to the second stage three to 12 weeks after the initial infection. In the second stage, also known as the early disseminated stage, patients present with a variety of symptoms such as a multifocal rash (secondary EM), fever, chills, fatigue, and joint stiffness. Neurological (Bell’s palsy, acute transverse myelitis, and meningitis) and cardiovascular (heart block and/or pericarditis) are manifestations of this second stage. The third stage, or the late disseminated stage, presents months to years after the initial infection with no definitive timeline and most commonly presents with recurrent asymmetric inflammatory arthritis [1-3].

Jamestown Canyon Virus (JCV) is an emerging arboviral pathogen transmitted to humans by mosquitos. The virus is found throughout the United States, most commonly in the Northeast and Upper Midwest surrounding the Great Lakes [4]. There are 10-75 cases reported annually in the United States with approximately half of the cases being neuroinvasive [4]. It is believed that there is an underrepresentation of overall infections based on under-diagnosis and under-reporting of less severe and asymptomatic cases. The incubation time can last from several days to two weeks. Symptomatic infections can present with fever, fatigue, headache, and meningitis [4]. Diagnosis relies on patient history and serologic testing of patient serum or cerebral spinal fluid (CSF).

With changes in climate, continued increase in travel, and increases in population, the spread of these disease vectors and the diseases themselves are on the rise [5]. A recent study has found around 26 species of mosquitoes can carry and transmit JCV [6]. Changes to climate may also lead to the spread of these vectors outside of their historical geographical areas, leading to an increased potential for overlap that did not, or rarely, existed previously. This can lead to an increase not only in the spread of singular diseases but also an increase in the possibility of co-infections.

## Case presentation

**Figure 1:**
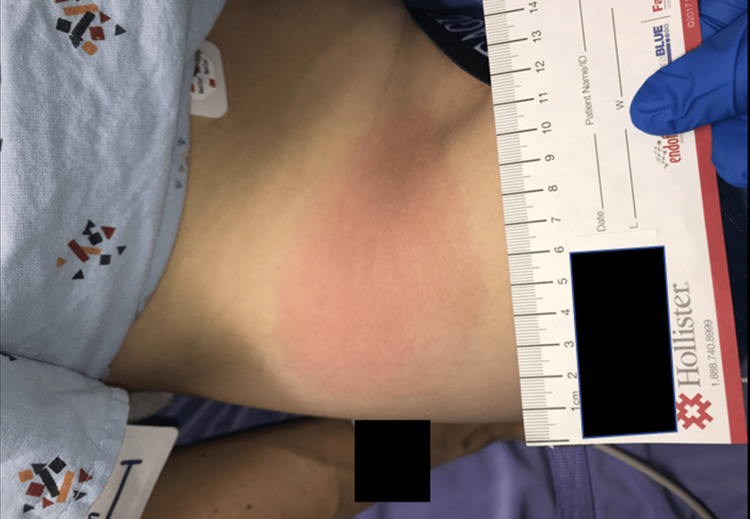
Erythema migrans on the patient’s abdominal right lower quadrant present upon admission

We present a 36-year-old man with a past medical history positive for generalized anxiety disorder, mild depression, allergic rhinitis, and herpes simplex labialis. His medications at the time of the emergency department (ED) visit were escitalopram 10 mg daily, fluticasone propionate inhaler 50 mcg as needed, lorazepam 1 mg as needed, and valacyclovir 1000 mg as needed. The patient did not smoke, or use any illicit drugs, and he drank socially. He denied any recent travels outside Wisconsin or any sick contacts. The patient presented to the ED with a five-day history of headache, fever, and nausea without vomiting. His headache was constant, frontal, and bilateral. He also reported new neck stiffness, diaphoresis, and chills. Upon further evaluation, he was found to have additional symptoms of fatigue, loss of appetite, recent unexpected weight loss, and mild photophobia. The patient was found to have tachycardia (120 beats per minute), fever (38.7^o^C), and a 15 x 10 cm circular erythematous rash, without central clearing, over the right lower quadrant of his abdomen (Figure 1). The patient denied any tick bites but reported significant outdoor activities. Initial blood work found increased levels of C-reactive protein (CRP) (109.2 mg/L), aspartate aminotransferase (AST) (145 U/L), alanine aminotransferase (ALT) (173 U/L), alkaline phosphatase (AP) (227 U/L), and bilirubin (1.3 mg/dL) (Table 1). Based on the erythematous rash, elevated liver function tests (LFTs), and CRP, he was started on IV (intravenous) doxycycline and IV ceftriaxone to cover for endemic tick-borne pathogens, mainly *Borrelia burgdorferi*, *Anaplasma phagocytophilum*, and *Ehrlichia chaffeensis*. A CSF sample was taken, which demonstrated nine nucleated cells and 50% neutrophils. CSF protein and glucose were normal. The patient was admitted for the further management of sepsis and possible Lyme disease. He was started on treatment with ceftriaxone and was given intravenous fluids, analgesics, and antiemetics.

**Table 1:** This table summarizes the patient’s basic workup chronologically from admission to two weeks following discharge. WBC, white blood cell; AST, aspartate aminotransferase; ALT, alanine aminotransferase

Laboratory Test	Day 1 (10/03)	Day 2 (10/04)	Day 3 (10/05)	Discharge (10/11)	2-Week Follow-Up (10/24)
Hemoglobin (g/dL)	14.6	13.6	13.6	14.9	14.9
WBC (mil/m^3^)	5.9	7.4	6.3	4.3	6.4
Neutrophils (mil/m^3^)	4.39	5.33	3.01	3.34	3.02
Lymphocytes (mil/m^3^)	0.80	1.32	2.39	2.27	2.69
Platelets (mil/mm^3^)	152	152	190	453	300
AST (U/L)	145	91	194	179	28
ALT (U/L)	173	136	228	511	64
Bilirubin (mg/dL)	1.3	0.6	0.5	0.7	0.4

**Figure 2:**
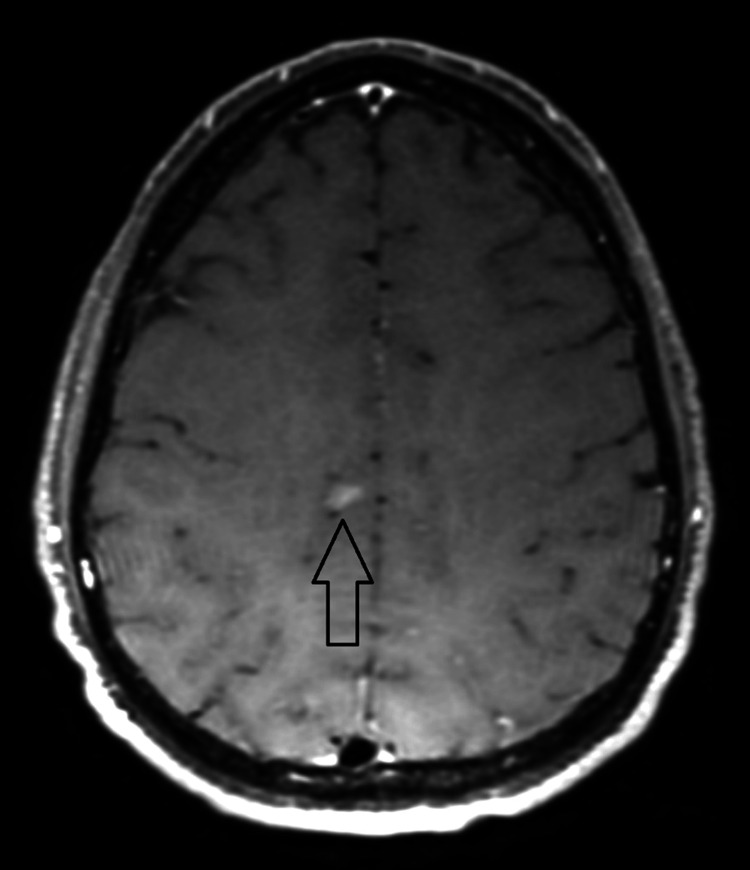
One of the two (nonspecific) enhancing brain lesions demonstrated by contrast-enhanced axial T1 weighted MRI

On day two, the erythematous rash had started to subside, with central clearing. An MRI was performed that presented two small indeterminate areas of vague enhancement, suggesting a possible inflammatory or infectious process (Figure 2). A Lyme polymerase chain reaction (PCR) of the CSF returned negative. In the following two days, they became afebrile and stated considerable improvement in headache, neck pain, and photophobia with the use of analgesics, antiemetics, and intravenous fluids. Additional tests from blood including *Ehrlichia/Anaplasma* PCR, *Babesia microti* PCR, Lyme disease immunoglobin M (IgM) and immunoglobin G (IgG) antibodies, and CSF testing for meningitis/encephalitis panel (Mayo Medical Laboratories) PCR panel all came back negative (Table 2).

**Table 2:** Illustrates extensive workup that was done on CSF and blood samples to elucidate the etiology of the patient’s encephalitis Ab - antibodies; CSF - cerebrospinal fluid; IgM - immunoglobulin M; IgG - immunoglobulin G; IGRA - interferon-gamma release assays; PCR - polymerase chain reaction; PRNT - plaque reduction neutralization test; VDRL - venereal disease research laboratory

Pathogen/Test	CSF	Blood
Gram stain	Negative	-
Cultures	Negative	Negative
Herpes simplex virus I and II PCR	Negative	-
Varicella zoster virus PCR	Negative	-
Cytomegalovirus PCR	Negative	-
Human parechovirus PCR	Negative	-
Human Herpesvirus 6 PCR	Negative	-
Epstein Barr virus PCR	Negative	-
Enterovirus 71 PCR	Negative	-
Adenovirus PCR	Negative	-
LCM virus IgM and IgG	Negative	-
West Nile virus IgM and IgG Ab	Negative	Negative
Jamestown Canyon virus IgM Ab	IgM positive, PRNT positive	-
St. Louis Encephalitis virus IgM and IgG Ab	Negative	-
Calif (LaCrosse) encephalitis virus IgM and IgG	Negative	-
West equine encephalitis virus IgM and IgG Ab	Negative	-
Human immunodeficiency virus PCR	-	Negative
East Equine Encephalitis Virus IgM and IgG Ab	Negative	-
Powassan virus IgM, IgG, PRNT	Negative	Negative
Escherichia Coli K1 PCR	Negative	-
Neisseria meningitidis PCR	Negative	-
Borrelia burgdorferi PCR, IgM, IgG Ab	Negative	Negative
Leptospirosis spp IgM and IgG	Negative	Negative
IGRA	-	Negative
Listeria monocytogenes PCR	Negative	Negative
Ehrlichia eauclairensis PCR	-	Negative
Ehrlichia chaffensis IgM and IgG	-	Negative
Anaplasma phagocytophilum IgG	-	Negative
Anaplasma phagocytophilum IgM	-	Negative
Anaplasma phagocytophilum PCR	-	Negative
Borrelia miyamotoi PCR	-	Negative
Streptococcus pneumoniae PCR	Negative	-
Streptococcus agalactiae PCR	Negative	Negative
Haemophilus influenza PCR	Negative	Negative
VDRL	-	Negative
Babesia microti PCR, IgG	-	Negative
Cryptococcus neoformans/gattii PCR	Negative	-

On day six, the erythematous rash had resolved, and his headache, neck stiffness, and pain were improving. During treatment with ceftriaxone, the patient developed ceftriaxone-induced liver injury and hepatocellular pattern, so ceftriaxone was discontinued and he completed treatment with doxycycline.

Three weeks following discharge, a serologic diagnosis of JCV was made with a positive CSF IgM antibody using enzyme-linked immunosorbent assay (ELISA) with confirmation by plaque reduction neutralization test (PRNT) performed at Wisconsin State Laboratory of Hygiene. One week later, repeat blood work was obtained, and it returned positive for *Borrelia burgdorferi *IgM antibodies. These results support the diagnosis of co-infection.

## Discussion

Meningitis is a medical condition defined by inflammation of the meninges or the outer covering of the brain [7]. Meningitis can be classified as either non-infective or infective. Infective meningitis can be further classified by its infective agent, i.e. (virus, bacteria, parasite, or fungus). Aseptic meningitis is a term used to describe meningitis in a patient with negative bacterial CSF cultures. Lastly, meningitis can be classified by its timeline, being either acute (lasting less than five days), subacute (lasting between five days and four weeks), or chronic (persisting over a period longer than four weeks) [8]. Meningitis can present with a wide array of symptoms, including headache, nausea, fever, chills, photosensitivity, and neck stiffness. It can often appear and progress quickly and is often associated with high morbidity and mortality. Lumbar puncture (LP) is used to identify the infective class, i.e. viral, bacterial, etc., based on values such as opening pressure, glucose, protein, white blood cell (WBC) count, and differentials [9]. Once the infective class is determined, targeted antimicrobial treatment is given.

The patient discussed here presented with many of the signs and symptoms typical for meningitis. He initially presented with a five-day history of headache, chills, neck stiffness, mild photophobia, and nausea, among other typical symptoms. Physical examination demonstrated EM, a pathognomonic rash of early localized LD. In someone who has a history of significant outdoor exposure and lives in an LD endemic area, EM is diagnostic of early localized LD. Erythema migrans usually appears within five to seven days of an infection and can last for up to three weeks [3]. Neuroborreliosis (manifested as meningitis) usually develops in the second stage (early disseminated LD), three to 12 weeks after the initial infection [3]. Infections that are not treated with antibiotics may have overlapping stages [10]. However, for both the erythema migrans and neuroborreliosis to be present at the same time is unlikely but not impossible. This unusual timeline prompted further investigation and led us to believe that the cause of this patient’s meningitis might be another pathogen, which proved to be correct.

JCV is an arbovirus (arthropod-borne virus) that belongs to the California serogroup together with the *La Crosse *orthobunyavirus, *Inkoo* orthobunyavirus, and snowshoe hare virus [4,11]. Similar to these viruses, JCV also is capable of causing neuroinvasive disease. In fact, while some infections might be asymptomatic, in patients who exhibit symptoms, JCV infection usually manifests as a febrile illness with headache, meningitis, and/or meningoencephalitis [11,12]. Unusual presentation mimicking migraine with aura and stroke has been reported as well [13,14]. JCV is an emerging cause of viral meningitis. Since arboviruses circulate in the bloodstream and CSF only transiently in the first few days following infection, isolating the viral RNA from these samples is not a very sensitive method for diagnosis. Diagnostics are based on serology from either blood or CSF [12]. A JCV infection can be diagnosed with ELISA as well as confirmatory tests like plaque reduction neutralization test (PRNT) or reverse transcription-polymerase chain reaction (RT-PCR). This testing is limited to a few public health laboratories in North America limiting their ability and turnaround time [15]. Due to significant cross-reactivity among the California serogroup, confirmation of positive serology is done by PRNT on the CSF sample [13].

In patients with viral meningitis, such as JCV meningitis, one would expect to find lymphocytic pleocytosis in CSF. However, CSF findings in patients with neuroinvasive JCV are various. In some cases of neuroinvasive disease CSF can be completely clear while in other cases, neutrophilic pleocytosis was demonstrated together with hypoglycorrhachia and increased proteinorachia mimicking bacterial meningitis [4,16]. Our patient had normal CSF protein levels and normal CSF glucose but only a slightly elevated white blood cell (WBC) count and an equal neutrophil and lymphocyte ratio. Our findings further add to the variety of CSF findings that can be seen with neuroinvasive JCV infection.

JCV has been found to affect all age groups with the majority of patients being immunocompetent individuals. While JCV in immunocompetent people is usually a self-limiting disease, and treatment is largely supportive, in immunocompromised patients, the clinical course might be more dramatic. A recent review of the literature showed that patients on rituximab (chimeric anti-CD-20 monoclonal antibody) might have a particularly prolonged course, and worse outcomes, largely due to their inability to mount appropriate serologic response [17]. In these patients, in addition to supportive care, ribavirin, steroids, and intravenous immunoglobulins were used with success [18]. Clinicians should be aware that rare cases of MOGAD (myelin oligodendrocyte glycoprotein antibody-associated disease) have been reported as a delayed autoimmune complication following JCV infection [19]. Lastly, is it important to understand this and other arbovirus’s role in co-infections. LD is commonly associated with co-infections with the likes of ehrlichiosis, anaplasmosis, and babesiosis, as all of them are transmitted through the same vector, the *Ixodes* tick. JCV is not commonly associated with co-infections, although a case of encephalitis with a JCV and varicella zoster virus (VZV) co-infection has been reported [20].

## Conclusions

Our patient contracted *Borrelia burgdorferi* through a tick bite and JCV through a mosquito bite. The overlapping geographical areas of these two vectors capable of transmitting infections to humans, and our patient’s significant outdoor exposure made this co-infection possible. This rare set of events, and clinical manifestations of both infections at the same time makes this presentation unique and quite challenging from a diagnostic perspective. Clinicians in areas that are endemic for tick-borne and mosquito-transmitted diseases need to maintain a high index of suspicion and be aware of the possibility of co-infection in order to make a timely diagnosis. Further research is needed to better understand the interaction of these pathogens with humans and to understand if co-infections lead to more severe clinical presentation.
